# Mechanism research and treatment progress of NAD pathway related molecules in tumor immune microenvironment

**DOI:** 10.1186/s12935-022-02664-1

**Published:** 2022-07-30

**Authors:** QinChen Xu, Xiaoyan Liu, Ghazal Mohseni, Xiaodong Hao, Yidan Ren, Yiwei Xu, Huiru Gao, Qin Wang, Yunshan Wang

**Affiliations:** 1 Department of Clinical Laboratory, The Second Hospital of Shandong University, 247 Beiyuan Street, 250033 Jinan, Shandong China; 2 Marine College, Shandong University, 264209 Weihai, China; 3 Department of Anesthesiology, Cheeloo College of Medicine, Qilu Hospital, Shandong University, 107 Wenhua Xi Road, Jinan, 250012 Shandong China

**Keywords:** NAD + metabolism, Tumor immune microenvironment, Tumor therapy, Immunotherapy

## Abstract

Nicotinamide adenine dinucleotide (NAD) is the core of cellular energy metabolism. NAMPT, Sirtuins, PARP, CD38, and other molecules in this classic metabolic pathway affect many key cellular functions and are closely related to the occurrence and development of many diseases. In recent years, several studies have found that these molecules can regulate cell energy metabolism, promote the release of related cytokines, induce the expression of neoantigens, change the tumor immune microenvironment (TIME), and then play an anticancer role. Drugs targeting these molecules are under development or approved for clinical use. Although there are some side effects and drug resistance, the discovery of novel drugs, the development of combination therapies, and the application of new technologies provide solutions to these challenges and improve efficacy. This review presents the mechanisms of action of NAD  pathway-related molecules in tumor immunity, advances in drug research, combination therapies, and some new technology-related therapies.

## Introduction

Nicotinamide adenine dinucleotide (NAD) was first discovered by British biochemists in 1906 and is defined as a substance in yeast that promotes fermentation reactions. NAD is a vital hydride receptor involved in the redox reaction, it can adjust hydrogenase active in various metabolic pathways, such as glycolysis, glutamine decomposition, and fatty acid oxidation, then it will go through the electron transport chain to generate ATP in eukaryotes. In addition, NAD is also used as a cofactor or substrate by hundreds of enzymes to participate in cell proliferation, differentiation, and functional regulation. In recent years, NAD has been proved to play an essential role in many diseases, including cancer [1].

The generation of tumors is closely related to immune cells. The interaction between tumor cells, immune cells, related cytokines, and various pathways constitute the tumor immune microenvironment, which can provide a favorable survival environment for cancer cells and inhibit immune cells from playing anti-tumor roles. In the tumor immune microenvironment, cancer cells characterized by malignant proliferation have an extreme demand for energy metabolism, which means it is necessary to accelerate the NAD cycle. For example, the Warburg effect can fully explain the characteristics of energy metabolism in the tumor microenvironment. So how do key enzymes in the NAD pathway affect the tumor immune microenvironment and play a role in tumorigenesis and cancer development? What is the current status of antitumor therapies based on these enzymes? This review links the key enzymes of the NAD pathway with the tumor immune microenvironment. Starting from the key enzymes promoting the production of NAD, NAMPT, NAD consuming enzymes, SIRT, PARP, and CD38. In this article, we show their mechanism in the tumor immune microenvironment in recent years, related drugs targeting these molecules and the progress of new therapies. We further discuss the limitations and development direction of relevant treatment methods.

## NAD metabolism and tumor immune microenvironment

**Fig. 1:**
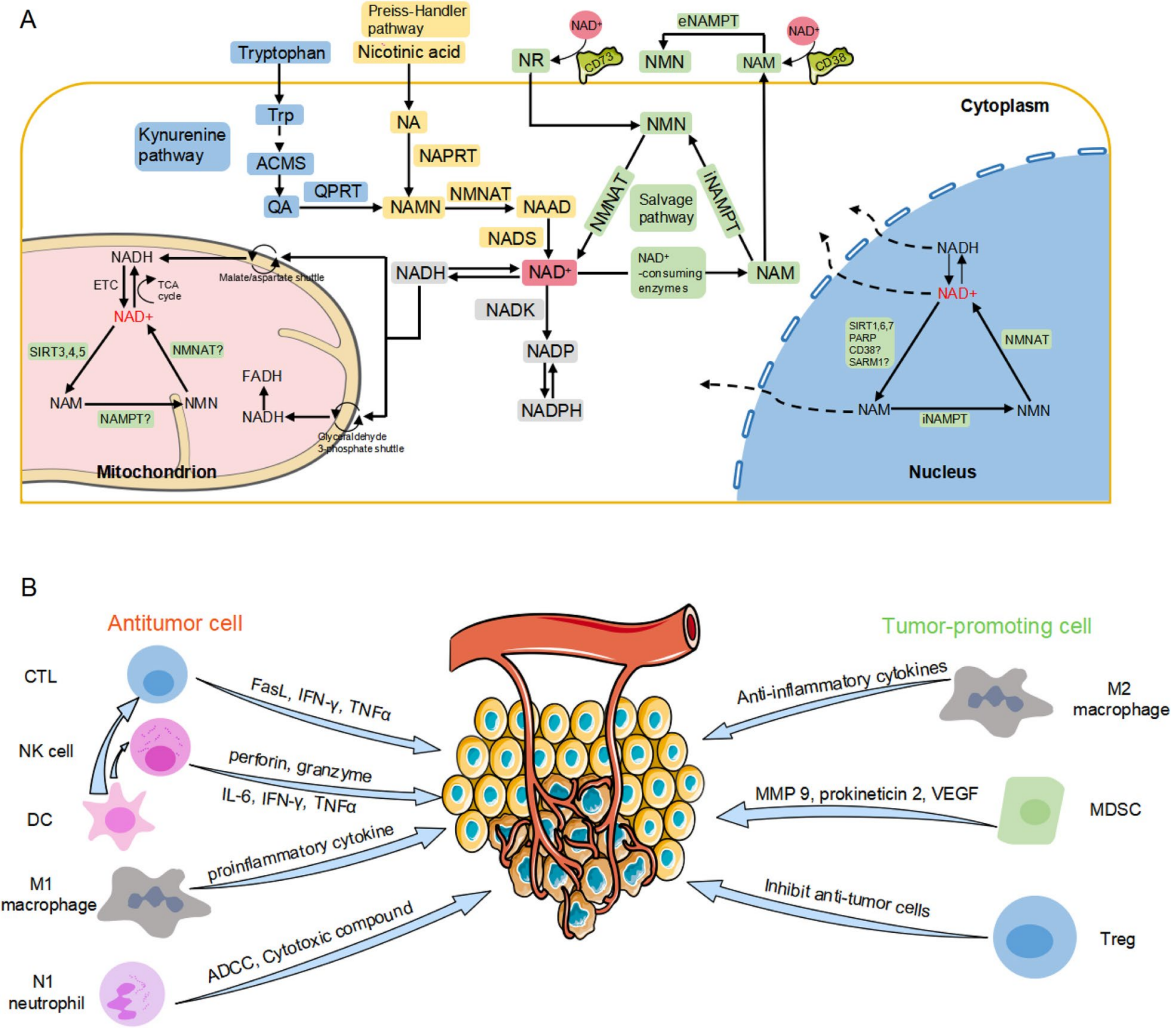
NAD + metabolism and TIME.** A** NAD + is synthesized by three pathways: Kynurenine pathway, Preiss-Handler pathway, and salvage pathway. Under the action of key synthetases such as NAMPT and consuming enzymes such as sirtuins, PARP, and CD38, NAD^+^ is continuously synthesized and consumed, which generates a large amount of ATP for the maintenance of cell life activities. **B** The cells in the tumor immune microenvironment mainly include anti-tumor cells and pro-tumor cells. The former mainly include cytotoxic T cells (CTLs), NK cells, dendritic cells (DC), M1 macrophages, and N1 neutrophils. The latter mainly includes M2 macrophages, bone marrow-derived inhibitory cells (MDSC) and regulatory T cells (Treg). These cells influence tumors through a variety of signaling pathways and cytokines

Mammals produce NAD + mainly by three pathways: de novo pathway (Kynurenine pathway), Preiss-Handler (PH) pathway, and salvage pathway (Fig. 1A). The intersection of the de novo synthesis pathway and the PH pathway is niacin mononucleotide (NAMN). The former uses tryptophan as the starting molecule and goes through a series of enzymatic reactions to produce quinolinic acid (QA), which is then converted to NAMN by quinolinic phosphoribosyltransferase (QAPRT/QPRT). The latter starts with niacin (NA) and generates NAMN under the action of nicotinate phosphoribosyltransferase (NAPRT). Finally, NAMN is catalyzed by nicotinamide mononucleotide adenylate transferase (NMNAT) and NAD synthase (NADS) to produce NAD^+^ [2, 3]. Nicotinamide mononucleotide (NMN) is generated by nicotinamide phosphoribosyltransferase (NAMPT) as the starting substance, and nicotinamide adenosine mononucleosyltransferase (NMNAT) generates NAD by transferring the adenosine portion from ATP to NMN [4, 5]. Warburg effect refers to the fact that glycolysis is dominant in the presence of oxygen, while oxidative phosphorylation is reduced, leading to the fermentation of pyruvate into lactic acid. This metabolic reprogramming of cancer cells is an adaptation to hypoxia and a key component of the malignant phenotype, which is considered a "marker of cancer" [6, 7]. The large amount of ATP produced by the Warburg effect requires cancer cells to maintain higher production of NAD, and NAD is also a central molecule required by tumor cells to support many key cellular processes [8]. It should be noted that the pathway of NAD production in cancer cells mainly depends on the salvage pathway or PH pathway, and the specific pathway depends on the normal tissues from the tumor. NAMPT and NAPRT, as rate-limiting enzymes in these two pathways, play a crucial role in the occurrence and development of cancer [9]. NAD-consuming enzymes can be divided into three categories: NAD hydrolases (CD38, SARM1), sirtuins, and PARPs. These enzymes use NAD as a substrate or cofactor and produce a byproduct, NAM, to recover NAD through the salvage pathway, so NAD is continuously synthesized, catabolized, and recycled in cells to maintain a stable intracellular level of NAD. In addition, these three enzymes also have a variety of critical cellular functions and play an indispensable role in the tumor immune microenvironment, which will be described in detail later.

Tumor immune cells in the immune microenvironment can be divided into two categories: promoting tumor immune cells and anti-tumor immune cells; these cells with complex communication networks secrete cytokines, chemokines and growth factors; these factors interact with tumor cells to form large and complex tumor immune microenvironment (Fig. 1B). The tumor-promoting immune cells were mainly composed of regulatory T cells (Tregs) and myeloid suppressor cells (MDSCs). Tregs are a double-edged sword in the human body. On the one hand, they can prevent autoimmune diseases caused by the overactivation of immune cells; on the other hand, their inhibitory function may prevent cytotoxic T cells (CTLs) from playing a role in cancer cells [10]. Myeloid suppressor cells can enhance angiogenesis by producing MMP9, kinetotropin 2, and vascular endothelial growth factor (VEGF), and also induce the migration of cancer cells to endothelial cells and promote metastasis [11]. At the same time, they disrupt major mechanisms of immune surveillance, including dendritic cell (DC) antigen presentation, T cell activation, M1 macrophage polarization, and natural killer (NK) cell cytotoxicity [12]. Tumor-associated M2-polarized macrophages promote aerobic glycolysis and anti-apoptotic properties of breast cancer cells in an extracellular vesicle-dependent manner [13].

Anti-tumor immune cells were mainly composed of CD8^+^ cytotoxic T cells, NK cells, DCs, M1-polarized macrophages, and N1-polarized neutrophils. CD8^+^ T cells can be induced to become CD8^+^ cytotoxic T cells when receiving antigen presented from DC, and the activated CTLs can kill the target cells through granular exocytosis and Fas ligand (FasL) mediated apoptosis. Interferon -γ (IFN-γ) and tumor necrosis factor α (TNFα) can also be secreted to induce cytotoxicity of cancer cells [14]. Activation and regulation of CTL require two signals: the first one is from the T-cell receptor (TCR) and the second one is from other receptors called immune checkpoints[15]. These immune checkpoints can be divided into two types: inhibitory checkpoints (e.g., CTLA-4 [16], PD-1 [17], TIM-3 [18, 19]) and stimulus checkpoints (e.g., ICOS [20, 21], OX-40 [22], CD27[23], and CD40L [24]). In some tumors, cancer cells inhibit the activation of CTLs by expressing ligands such as programmed death ligand-1 (PD-L1) that bind to inhibitory checkpoints [25], which is considered an important mechanism of tumor immune escape. NK cells function similarly to CD8^+^ T cells; DCs can secrete chemokines to induce their migration into cancer tissues [26]. NK cells mediate tumor-killing response mainly by releasing perforin and granzymes to induce apoptosis of target cells [27]. In addition, NK cells can secrete pro-inflammatory cytokines and chemokines (such as IFN-γ, TNF, IL-6, GM-CSF, and CCL5) to promote antitumor activity. As mentioned above, DCs function primarily as professional antigen-presenting cells (APCs), which present antigens, provide costimulatory signals for T cell activation, and help immune-effector cells gather in tumors. Classically activated macrophages M1 produce pro-inflammatory cytokines and reactive oxygen species/nitrogen substances essential for host defense and tumor cell killing [28].

## The mechanism of NAD pathway related molecules in TIME

### NAMPT

Cancer cells require high amounts of energy to support their proliferation, implying an increased demand for NAD through NAMPT overexpression to fund cellular metabolism and NAD depletion responses [29]. NAMPT, as a rate-limiting enzyme in the salvage pathway, has extracellular and intracellular forms. The intracellular form (iNAMPT) is located mainly in the cytoplasm and is the basis of cell maintenance of energy metabolism, and its inhibition can lead to ATP depletion [30–32]. The extracellular form (eNAMPT) was initially named PBEF (Pre-B enhancer) because of its ability to regulate B cell differentiation when combined with stem cell factors and IL-7 [33]. ENAMPT can be secreted by neutrophils, macrophages, and other cells under the induction of cellular stress and inflammatory response and acts as a protein molecule to activate various intercellular signaling pathways such as immune cells and cancer cells [8]. ENAMPT also promotes cancer by regulating the tumor microenvironment, enhancing tumor metabolism, and promoting epithelial-mesenchymal transformation (EMT) [34, 35].

**Fig. 2:**
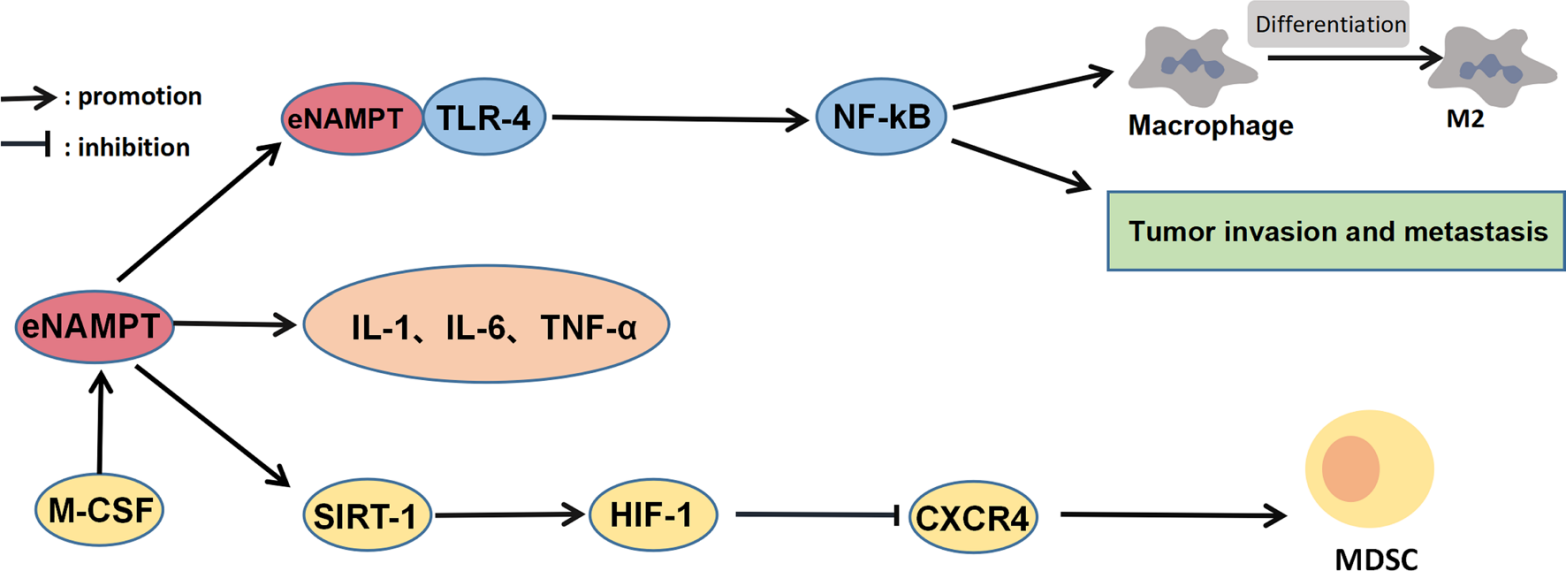
NAMPT in TIME. In the tumor immune microenvironment, eNAMPT can regulate the production of cytokines such as IL-1, IL-6, and TNF-α, inhibit anti-tumor immunity, promote tumor invasion and metastasis through M-CSF/SIRT-1/HIF-1/CXCR4 and TLR-4/ NF-KB

Elevated NAMPT expression has been widely reported in many solid and hematological malignancies, including pancreatic cancer [36], gastric cancer[37], glioma [38], melanoma [39], and lymphoma [40]. Some studies have shown that NAMPT is a cancer-related factor and plays a vital role in the development and metastasis of tumors along with some immune-related substances. It also has been shown that NAMPT overexpression and exogenous eNAMPT induced EMT in breast cancer cell lines [41] and eNAMPT promoted osteosarcoma cell migration and invasion[42]. Based on extensive studies of inflammatory preclinical models of disease [43–46], it is speculated that eNAMPT may accelerate tumor invasion through its function as damp-related molecular pattern protein (DAMP) in response to hypoxia and mechanical stress[47] and activates toll-like receptor 4 (TLR4) binding. TLR4 connectivity significantly increases NF-kB-dependent expression of inflammatory chemokines and cytokines that regulate cell growth/survival and angiogenesis [43, 48]. At the same time, eNAMPT/ TLR4-mediated inflammatory signaling may contribute to the production of tumor-supporting M2 macrophages, which are thought to be involved in tumor occurrence, recurrence, invasion, and metastasis [48, 49], while the use of the immunomodulator lenalidomide can reverse M2 polarization and significantly reduce NAMPT expression in Chronic Lymphocytic Leukemia (CLL) lymphocytes [49]. NAMPT is part of the M-CSF/NAMPT/SIRT1/HIF-1/CXCR4 axis that controls the mobilization of bone marrow mesenchymal stem cells. Macrophage colony-stimulating factor (M-CSF) increases the myeloid cell level of NAMPT. NAMPT inhibits CXCR4 by SIRT1-mediated inactivation of HIF-1-driven CXCR4 gene transcription, thereby mobilizing immature MDSC to produce inhibitory nitric oxide (NO), while drug inhibition of NAMPT or myeloid-specific ablation can block MDSC mobilization (Fig. 2). Reactivation of specific antitumor immunity, reversal of tumor immunosuppression, and restoration of clinical efficacy of immunotherapy in cancer patients [50]. However, NAMPT is not a complete oncogenic factor and can interact with sirtuins to regulate cell function [51, 52], its tumor-suppressive effect may be related to this. For example, a team studied the contribution of NAMPT to tumor-associated neutrophils tumorigenic transformation. They found that NAMPT inhibited the tumorigenesis of neutrophils by inhibiting SIRT-1 signal transduction and then blocked the transcription of angiogenic genes to play an anti-tumor role [53]. However, compared with the direct function of NAMPT, this effect is weakened, and NAMPT is still a tumor-causing factor in general.

**Fig. 3:**
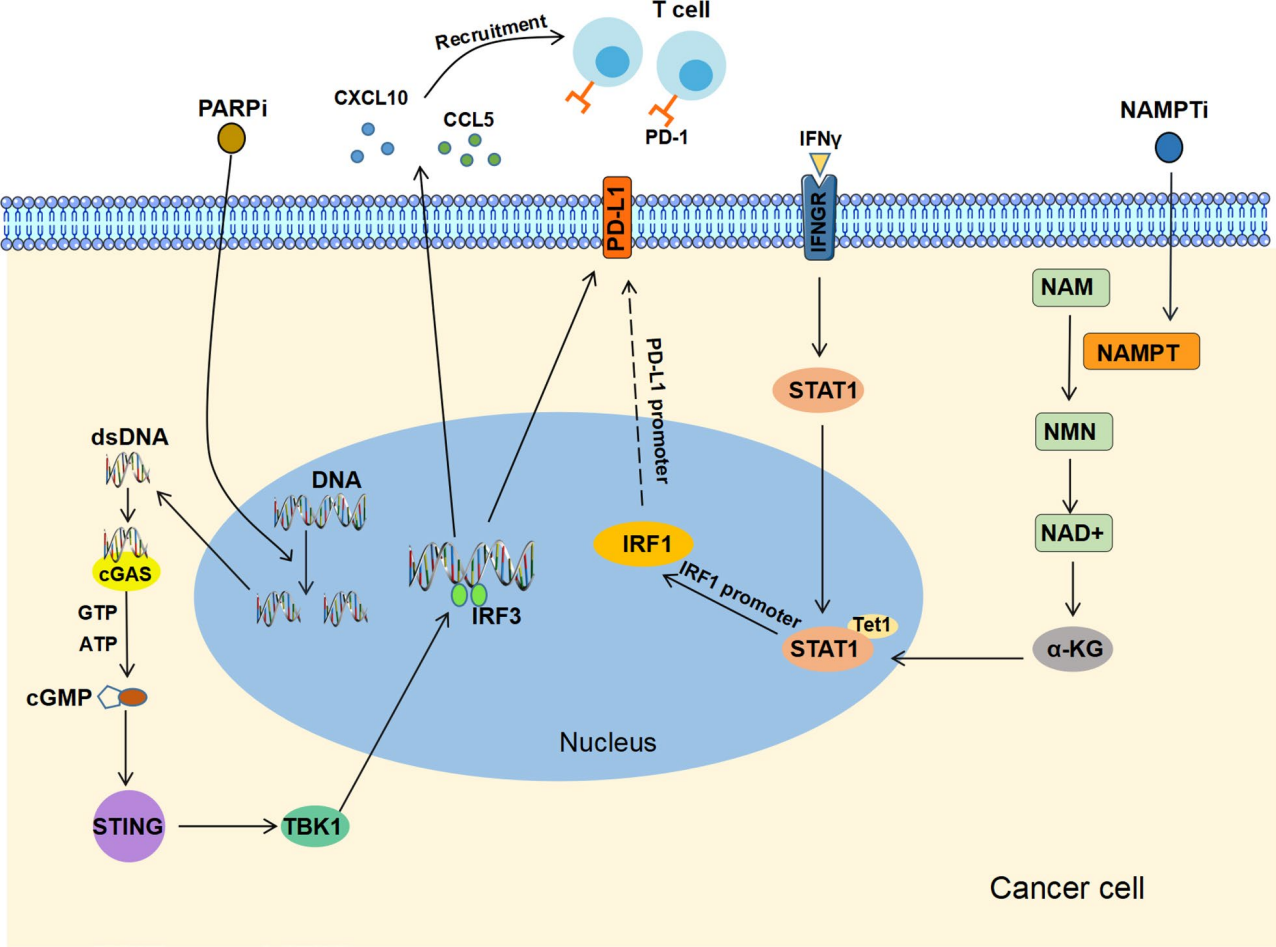
NAMPTi, PARPi and immunotherapy. Under IFN-γ stimulation, JAK/STAT/ interferon regulatory factor-1 (IRF1) signal axis positively mediates PD-L1 expression. Inhibition of NAMPT can reduce the expression of downstream STAT1 molecules, indicating the prospect of NAD complementary therapy and immune checkpoint blocking therapy. Under the action of PARP inhibitors, the content of dsDNA in the cytoplasmic is increased, which activates the cGAS/STING pathway and leads to the generation of type 1 interferon (IFN) reaction, which on the one hand, enhances the relevant chemokines and leads to the accumulation of T cells; on the other hand, increases the expression of PD-L1 and leads to the depletion of T cells. This adverse effect can be eliminated by PD-1/PD-L1 blockade

In the tumor immune microenvironment, NAMPT interacts with inflammatory factors, and many previous studies have shown that extracellular NAMPT promotes the production of many inflammatory factors, such as IL-1α, IL-1β, IL-6, and TNF-α[54, 55]. In turn, NAMPT is a target of signal transducer and activator of transcription 1 (STAT1) during cell activation by IFN-γ, which is an essential driver of macrophage polarization and antitumor response. The expression of PD-L1 was positively mediated by the JAK/STAT/ interferon regulatory factor-1 (IRF1) signal axis under IFN-γ stimulation (Fig. 3). STAT1 and its downstream molecule IRF1 were significantly decreased after NAMPT silencing under IFN-γ stimulation, while p-STAT1 was increased in NMN-supplemented cells. NAD metabolism regulates PD-L1 induction in tumors through the STAT1-dependent IFN-γ signaling pathway, suggesting that NAD^+^ complementary therapy combined with an anti-PD-1 /PD-L1 antibody regimen may be a novel therapeutic strategy [56]. Interestingly, the level of NAD^+^ in tumor-infiltrating lymphocytes is relatively low, although the Warburg effect increases the levels of NAD^+^ in tumor cells. In line with this, the level of NAMPT in tumor-infiltrating lymphocytes (TILs) is also low, which leads to inhibition of the glycolysis pathway and destruction of mitochondrial function, thereby blocking ATP synthesis and TCR cascade signaling. This mechanism leads to impaired metabolism and loss of the antitumor ability of TILs [57]. In conclusion, NAMPT can regulate the expression of the surface protein of immune cells in TIME through indirect signal transduction and can also affect the energy metabolism of immune cells through direct metabolic regulation. These newly discovered mechanisms lay a foundation for applying NAMPTi to tumor immunotherapy.

### Sirtuins

Sirtuins are a class of deacetylases or ADP-ribosyltransferases that can use NAD as a cofactor to remove acetyl groups from acetylated proteins and produce nicotinamide (NAM) and acetyl metabolites. Seven deacetylases SIRT1-7 have been identified in mammals, and their common structural characteristics are the highly conserved 275 amino acid catalytic core domain and NAD binding domain, while differences exist in the N and C terminal domain related to cell localization and target specificity [58]. SIRT-1, SIRT-6, and SIRT-7 exist in the nucleus, while SIRT-2 mainly exists in the cytoplasm. SIRT-3, SIRT-4, and SIRT-5 exist in the mitochondria.

**Fig. 4:**
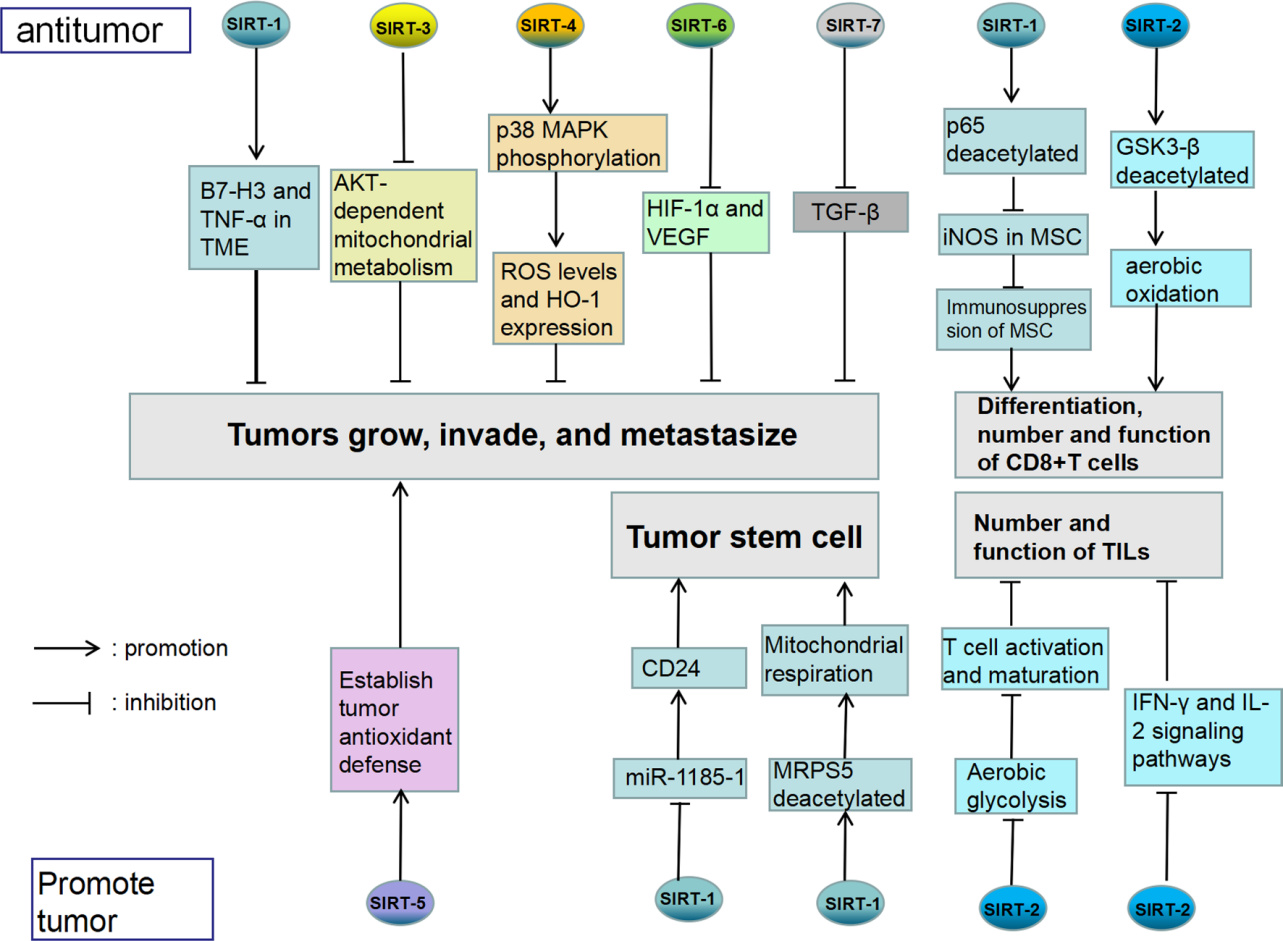
Examples of the role of sirtuins in tumors. SIRT-1/2/3/4/6/7 can play an anti-tumor role, and SIRT-1/2/5 can play a pro-tumor role

SIRT family is a family of proteins with extensive distribution and complex functions. Its tumor suppressor or protumor effect depends on different tumor types, targeted proteins, and signaling pathways within the same tumor. SIRT-1/2/5 could promote the development of the tumor (Fig. 4). In lung adenocarcinoma models with tumor suppressor gene defects, SIRT-1 overexpression can make cancer cells more dependent on mitochondrial oxidative phosphorylation and enrich cancer stem cells which leads to epidermal-growth-factor-receptor and tyrosine-kinase-inhibitor (EGFR-TKI) resistance [59], SIRT-1 also enhances mitochondrial respiration and glycolytic protein production by increasing the acetylation of mitochondrial ribosomal protein S5 (MRPS5) in the mitochondria and nucleus, thereby contributing to the Warburg effect, which is conducive to the maintenance of stemness in HCC stem cells, leading to poor prognosis [60]. In addition, SIRT-1 promotes CD24 expression by inhibiting the expression of the new tumor suppressor miR-1185-1 through histone deacetylation, thereby increasing the stemness and invasiveness of colorectal cancer cells [61]. Inhibition or deficiency of SIRT-2 enhances aerobic glycolysis; oxidative phosphorylation during T cell activation and maturation increases fatty acid oxidation to enhance TIL persistence and up-regulates IFN-γ response and IL-2 signaling pathways, which stimulates T cell proliferation and effector function. These effects suggest that SIRT-2 inhibition may trigger the antitumor activity of T cells [62]. The tumor-promoting effect of SIRT-3 is mainly related to the maintenance of cancer stem cells. For example, the synergistic interaction of mitochondrial chaperone tryptophan RNA-binding attenuation protein 1 (TRAP1) and SIRT-3 in glioma stem cells (GSC) increases mitochondrial respiration and reduces the production of reactive oxygen species. Thus maintaining the dryness of GSC and its ability to adapt to the nutritionally deficient environment [63]. Meanwhile, studies have also shown that SIRT3 promotes the chemical resistance of acute myelocytic leukemia (AML) by regulating mitochondrial metabolism [64]. SIRT-5 is not essential for normal cells but plays an important role in tumor cells because cancer cells rely on SIRT-5 to establish crucial cellular antioxidant defenses, and studies have shown that SIRT-5 inhibitors can delay tumor growth and metastasis, suggesting that SIRT-5 may be an important therapeutic target [65].

SIRT-1/2/4/6/7 acts as a tumor suppressor mainly through deacetylation (Fig. 4). In the liver metastasis model of colorectal cancer, SIRT-1 attenuates the immunosuppressive ability of MSC and enhances its pro-inflammatory ability by inhibiting the expression of inducible nitric oxide synthase (iNOS) in mesenchymal stem cells by deacetylating p65, thereby increasing the number of CD8^+^ T cells to enhance local immunity and inhibit tumor development [66]. Inhibition of SIRT-1 upregulates B7-H3 and TNF-α in the tumor microenvironment, thereby inducing tumor immune escape [67]. SIRT-1 can inhibit Th17 differentiation by deacetylating STAT3, thereby slowing tumor growth. SIRT-1 activator metformin can also limit harmful Th17 amplification by reducing IL-17 A and exerting an anti-tumor effect [68]. It has also been confirmed that SIRT-2 can mediate the differentiation of CD8^+^ T cells by inhibiting glycogen synthase kinase-3β (GSK-3β) acetylation and promoting aerobic oxidation in breast cancer patients. In contrast, CD8^+^ T cells and NK cells can effectively eliminate tumor cells in breast cancer, suggesting that SIRT-2 may also be a tumor suppressor factor [69]. Previous studies have shown that SIRT-3 can mediate metabolic reprogramming by down-regulating HIF-1 through deacetylation, thus reducing glycolysis rate to inhibit the Warburg effect in tumor cells [70]. In recent years, it has also been found that SIRT-3 can inhibit protein kinase B-dependent mitochondrial metabolism and EMT and play an anti-tumor role [71]. SIRT-4 can regulate the phosphorylation of P38 mitogen-activated protein kinase (MAPK) to control reactive oxygen species (ROS) level and heme oxygenase-1 expression, change the tumor hypoxia microenvironment, promote the apoptosis of cancer cells, and play an anti-tumor effect [72]. In lung cancer models, SIRT-6 overexpression may decrease HIF-1α and VEGF expression and promote prolyl hydroxylase-2 expression, thereby inhibiting angiogenesis and tumor growth [73]. In breast cancer models, SIRT-7 deficiency activates transforming growth factor-β (TGF-β) signaling and enhances EMT, and resveratrol activates SIRT-7 deacetylase activity, inhibits lung metastasis of breast cancer, and improves survival rates [74].

There are many types of sirtuins, each of which is involved in complex signaling pathways, so it is difficult to discuss the function of sirtuins apart from specific tumor types. In the same type of tumor, different sirtuins may play different roles. For example, in the lung cancer model mentioned above, SIRT-1 can enrich tumor stem cells, while SIRT-6 overexpression can inhibit the generation of tumor blood vessels. The same sirtuins also have different effects in different tumors. For example, SIRT-1 is beneficial to maintain the dryness of liver cancer stem cells, while it can help restore anti-tumor immunity in TIME in the liver metastasis model of rectal cancer. In summary, we should specifically analyze the effects of sirtuins on tumors based on objective experimental conclusions, which is particularly noteworthy in the research, development and indications of sirtuins-related drugs.

### PARPs

In mammals, there are 17 kinds of poly (ADP-ribose) polymerases (PARP) known as PARP1-16 (PARP5 is divided into A and B). PARP can catalyze the cleavage of β-NAD^+^ and transfer the ADP-ribose portion to specific amino residues of the receptor protein. The process is called ADP-parylation, and the formation of poly (ADP-ribose) polymers affects the structure and function of target proteins. PARP is expressed in many tissues and cells and is involved in DNA repair, transcriptional regulation, signal transduction, and metabolic regulation. It is also related to cancer, diabetes, neurodegeneration, and other diseases [75]. Here we mainly introduce the most studied and important functions of PARP-1 and PARP-2 in immune regulation.

Previous studies have shown that PARPs can regulate a range of cytokines, such as Th1 cytokines (IL-2, IFN-γ), Th2 cytokines (IL-4, IL-5, Il-10), TGF-β, and chemokines CXCL10, CCL5, CCL4 and CCL9 [76–78]. In addition, PARP also participates in tumor cell proliferation and EMT through co-activation of NF-κB and MAPK [79, 80]. In the immune microenvironment, the synergistic action of PARP-1 and PARP-2 can prevent the accumulation of unrepaired DNA breaks during T cell proliferation and response to antigen, thus avoiding T cell death and facilitating the differentiation of naive T cells into CD4^+^ and CD8^+^ T effector cells to perform their functions [81]. The activated T nuclear factor (NFAT) is an important transcription factor in T cell development and function. The inhibition of PARP-1 regulates the activity of NFAT through the PARylation of PARP, resulting in increased NFAT-dependent trans-activation [82]. In MC-38-induced colon cancer models, PARP-1-deficient mice showed accelerated tumor growth and reduced Th1 and CD8^+^ T cell response, suggesting that PARP-1 in macrophages regulates Th1 cell response to tumor [83]. Overexpressed PARP-1 enhances tumor angiogenesis by upregulating VEGF, thereby promoting tumor growth and metastasis [60]. Transcription cofactor PARP-1 also helps leukemia stem cells (LSCs) evade immune surveillance by down-regulating natural killer cell-activated receptor ligand [84]. Interestingly, single and double PARP-1 and PARP-2 deficiency play opposite roles in the tumor microenvironment. The double deficiency of PARP-1/PARP-2 reduces the number of infiltrating T and NK cells in the microenvironment, creating an environment conducive to tumor growth. A single defect in each protein limits tumor progression [85]. Therefore, as a cell signal regulator, the role of PARP in TIME is difficult to be fully defined, but its activation of tumor-associated immune cells can be considered for tumor immunotherapy. If they are used together, it should also be noted that the promotion of tumor blood vessels and stem cells should not be ignored for a particular type of tumor. Finally, we would like to emphasize that PARPs are still thought to be primarily tumor-promoting due to their essential role in DNA repair, which we will discuss later in this article.

### CD38

CD38, thought initially to be a cell surface marker on thymocytes and activated T cells, has now been found to be highly expressed in various immune cells (e.g., B cells, macrophages, DC, NK cells) but constitutively expressed in most tissues. As a major NAD-consuming enzyme, it is crucial for maintaining NAD homeostasis [86–88]. CD38 is a single-chain glycoprotein with a single transmembrane segment, which can be topologically expressed as extracellular type II or intracellular type III membrane protein with a catalytic domain based on its membrane direction[89]. CD38 catalyzes the cycle-conversion of NAD into cyclic ADP-ribose (cADPR) and hydrolyzes cADPR to form ADP-ribose (ADPR) [82]. ADPR and cADPR act as second messengers and control various cellular functions through Ca^2+^ mobilization [90, 91].

It is generally believed that the high expression of CD38 is associated with several hematological malignancies, which is an important feature of multiple myeloma and is also associated with B-cell chronic lymphoblastic leukemia, acute myeloid leukemia, acute lymphoblastic leukemia, and acute promyelocytic leukemia [92]. High expression of CD38 on the relevant cell surface in TIME induces an immunosuppressive microenvironment that inhibits effector T cell function and promotes tumor immune escape by promoting tumor angiogenesis, such as in multiple myeloma. High expression of CD38 can promote the production of immunosuppressive MDSC and regulatory T cells [93]. In chronic lymphocytic leukemia, CD38 overexpression promotes the secretion of VEGF and anti-apoptotic protein MCL-1 by CLL cells, both of which are associated with a poor prognosis of CLL. It has also been reported in recent years that lymphoid and bone-marrow-derived immune cells present at the site of solid tumors exhibit high expression of the CD38 cell surface, which is negatively correlated with the prognosis of the disease [94]. For example, studies have shown that increased expression of CD38 helps prostate cancer cells exhibit stem-like characteristics [95] and helps CD133 + CXCR4 + lung cancer stem cells evade immune surveillance [96]. The possible mechanism is that cADPR, the main hydrolysate of CD38, induces the opening of the iron channel of TRPM2, leading to intracellular Ca^2+^ influx, which then leads to increased Nuclear Factor erythroid 2-Related Factor 2 (NRF2) level and decreases Kelch-like ECH associated protein-1 (KEAP-1) expression in lung cancer, thus accelerating tumor progression. This suggests that selectively targeting the enzyme activity of CD38 in the tumor microenvironment may be a strategy for treating solid tumors [97].

## NAD pathway-related tumor therapy

### NAMPT inhibitor

Several previously identified NAMPT inhibitors have shown efficacy in cancer models, including ovarian, colorectal, prostate, pancreatic, and non-small cell lung cancers, neuroblastoma, and fibrosarcoma [98]. NAMPT inhibitors FK866, GMX1778 (CHS828), and GMX1777, a precursor of GMX1778, were evaluated in clinical trials in patients with advanced solid tumors, but these agents did not exceed Phase II due to severe toxicity, including thrombocytopenia [99, 100]. The dose-related toxicity of NAMPTi also limits its clinical application. Hematological toxicity caused by high-dose administration is the most significant side effect in patients treated in phase I and II solid tumor trials, of which thrombocytopenia is the most common. Others include anemia and neutropenia [101, 102]. Related preclinical studies have suggested that retinotoxicity and cardiotoxicity may also be potential side effects of NAMPTi [103, 104]. Nevertheless, targeting NAMPT remains an attractive strategy that is being explored by multiple drug development teams.

Solving the problem of the toxicity of NAMPTi to normal cells will make NAMPTi more useful in clinical trials. Firstly, we should innovate on the physical and chemical characteristics of NAMPTi itself. For example, a team discovered a new NAMPT inhibitor OT-82, which has great potential in treating hematological malignant tumors. Moreover, in toxicological studies in mice and non-human primates, OT-82 showed no cardiac, neurological, or retinal toxicity compared with other NAMPT inhibitors [105]. Secondly, it can also be combined with some adjuvant drugs to reduce cytotoxicity: Zhao, Genshi et al. reported that NAMPTi, named LSN3154567, inhibits the proliferation of many different cancer cell lines, especially NAPRT1-deficient cancer cell lines. When combined with folic acid, NAMPTi can avoid retinal and hematological toxicity. It is expected to be a novel anticancer drug [106]. The limited efficacy of NAMPT inhibitors is partly due to their failure to interfere with the non-enzymatic function of NAMPT. Ying Wu et al. have constructed a novel NAMPT inhibitor PROTAC A7 using Proteolysis-Targeting Chimera (PROTAC) technology. This compound can bind E3 ubiquitin ligase to NAMPT, causing NAMPT to be ubiquitinated and degraded by the ubiquitin-proteasome system. This method can effectively intervene in the non-enzymatic activity of NAMPT and reduce iNAMPT and eNAMPT. Thus inhibiting the expansion of MDSCs from restoring anti-tumor immunity [107].

Because of the unique and extensive effects of NAMPT in cells, the development of inhibitors with dual targeting functions can improve the anti-tumor efficacy and reduce the side effects caused by the drug alone, which is a promising therapeutic strategy. KPT-9274 is a dual inhibitor of PAK4/NAMPT, which mainly destroys the binding of nicotinamide substrates and the functional structure of NAMPT to exert the inhibitory effect of NAMPT. Animal experiments have shown that it can effectively induce AML cell cycle arrest and apoptosis, thus inhibiting the proliferation of AML cells. It has preferential toxicity to leukemia cells and lower toxicity to normal hematopoietic cells. KPT-9274 is undergoing clinical trials in solid tumors and non-Hodgkin’s lymphoma. Further studies are needed to identify the subset of patients who benefit from treatment with KPT-9274 and the associated combination strategy [108]. Compound 35, a highly potent NAMPT/HDAC dual inhibitor, is reported to have good and balanced activity against NAMPT and histone deacetylase (HDAC1) and can effectively induce apoptosis and autophagy, ultimately leading to cell death. Moreover, compound 35 has shown an excellent in vivo antitumor effect in the colon cancer xenograft model. Although its thrombocytopenia side effects and advantages over NAMPTi and HDACi combination therapy remain to be verified, it provides an effective strategy for developing multi-target antitumor drugs as a lead compound for developing new drugs [109]. Based on the NAMPT inhibitor CHS828 and EGFR inhibitor erlotinib, a compound 28 lacking the Michael receptor with a good double inhibitory effect were screened through the fusion of pharmacophore to inhibit the proliferation of several cancer cell lines in vitro. Moreover, it showed significant in vivo antitumor efficacy in the nude mouse model of human non-small cell lung cancer (NSCLC) (H1975) xenotransplantation [110].

Whether tumor cells depend more on NAMPT or NAPRT is associated with the normal tissue from which the tumor originates [9], which was mentioned in the previous paper. The expression mechanism and interaction of NAPRT and NAMPT in cells indicate that: For different types of tumors, the selection of appropriate NAMPT or NAPRT inhibitors will have better effects, and the combination of NAPRTi and NAMPTi is also a possibility. Many studies have shown that tumors are more sensitive to NAMPT inhibitors and have better effects in cells expressing absent or silenced NAPRT [40, 111, 112]. For example, in most EMT subtypes of gastric tumors, the loss of NAPRT expression makes cancer cells more dependent on NAMPT to produce NAD^+^. Studies have shown that NAMPT inhibitor FK866 has great potential in these refractory tumors [113]. Notably, when NAMPT is depleted in salvage-dependent tumors, they can still maintain NAD supply through the alternative salvage pathway, and the dual gene inhibition of NMRK1 and NAMPT leads to more effective NAD reduction and significant tumor suppression in vivo [9].

### Sirtuins activator and inhibitor

Resveratrol, a natural preparation that has been discovered and widely studied, is a plant antitoxin, which can participate in the regulation of relevant cell signaling pathways, and thus play an anticancer role by promoting the expression of anticancer factors, inhibiting the proliferation of tumor cells and inhibiting tumor angiogenesis [114]. It is the first polyphenol reagent recognized as an activator of SIRT-1/3/7 [115], whose cytotoxic effects on breast cancer cells depend on SIRT-1 [116]. Other bioactive ingredients also have anticancer effects, such as SIRT-1 activator salvianolic acid B [117, 118] and SIRT-3 activator Oroxylin A [119]. In addition, melatonin can enhance the activity of the pyruvate dehydrogenase complex by stimulating SIRT-3, thereby enhancing mitochondrial energy metabolism to reverse the Warburg effect. This change significantly inhibits the proliferation of lung cancer cells, revealing the potential anticancer use of melatonin [120]. In addition, it has been reported that atlodes atlodes, a traditional Chinese medicine, can inhibit the occurrence and development of glioblastoma multiforme (GBM) by up-regulating SIRT-3, but further clinical verification is still needed [121].

Sirtuins are one of the main proteins that regulate cancer stem cells and EMT and drive tumor resistance [122]. In recent years, many SIRT modulators have been developed to improve drug resistance of related tumor chemotherapy drugs. For example, SIRT-1 inhibitor AMurensin G reduced recombinant forkhead box protein O1 (FoxO1) and multidrug resistance 1 (MDR1) protein levels in doxorubicin-resistant breast cancer cells [123], Psammaplin A can also overcome multidrug resistance of breast cancer cells by inhibiting SIRT-1 and inducing autophagy [124]. Lead compounds 2800Z and 40569Z can specifically inhibit the deacetylation activity of SIRT-7 in vitro and in vivo, prevent proliferation, enhance the chemical sensitivity of sorafenib, and reduce tumor burden. The mechanism may be related to SIRT-7 /p53/NOXA axis [125].

A team reported an effective SIRT-6 activator, MDL-811, which activates SIRT-6 in an allosteric manner and is more selective than other HDACs. MDL-811 showed high antitumor activity both in cytological experiments and in vivo models. Further studies showed that member 1 of 24 subfamily A of the cytochrome P450 family was a new target gene of SIRT-6 to inhibit colorectal cancer (CRC) proliferation, MDL-811, and vitamin D3 could synergistically enhance the antitumor efficacy. This provides new ideas for developing a wide range of therapeutic agents for CRC [126]. Another SIRT-6 allosteric activator, MDL-800, has also been shown to inhibit the proliferation of 12 NSCLC cell lines in a dose-dependent manner. More importantly, MDL-800 can enhance the anti-tumor effect of EGFR-TKI to improve drug resistance in Osimertinib-resistant NSCLC cells. MDL-800 is promising as a lead compound in NSCLC alone or combination with EGFR-TKI [127]. Using molecular docking and molecular dynamics simulations, a team identified three plant-derived sirtuins inhibitors, sulforaphane, kamanol, and apigenin, which have good inhibition against SIRT-1/3/5/6 and can be used as potential drug candidates against multiple deacetylases [128]. Sosbo is a SIRT-1-specific benzoxazine inhibitor screened from 170,000 compounds. It is more efficient and selective than Selisistat (EX527), permeates cell membranes and has no significant toxicity [129]. These studies suggest that advances in the development of specific pharmacologic sirtuins-related drugs are critical to understanding the potential of sirtuins activators or inhibitors in cellular function and clinical outcomes.

In recent years, the research and development of SIRT family-related drugs have mainly focused on the innovation of drug structures. For example, a class of compounds based on the structure of 1,2, 4-oxadiazole have been shown to have considerable anti-proliferative activity in leukemia cell lines. Changes in α-tubulin acetylation levels suggest that this anticancer activity may be achieved by inhibiting SIRT-2 [130]. Another novel N-(3-(phenoxy methyl) phenyl) acetamide derivative also inhibits NSCLC cell proliferation by selectively inhibiting SIRT-2 [131]. (R) -40-methylKlavuzon and its derivative TK126 have cytotoxic effects on cancer stem cells in the huh-7 hepatocellular carcinoma cell line and showed more effective extracellular toxicity compared with sorafenib and regorafenib, possibly due to inhibition of SIRT-1/HDAC and CRM1 activity leading to cell cycle block [132]. The selective SIRT-6 activator 2- (1-benzofuran-2-yl) -N- (diphenylmethyl) quinoline-4-formamide (12Q) is also a promising lead compound for the treatment of pancreatic ductal adenocarcinoma [133].

### PARP inhibitor and the possibility of immunotherapy in combination

Currently, four PARP inhibitors (PARPi), Olaparib, Rucaparib, Niraparib, and Talazoparib have been approved for the treatment of breast, pancreatic, prostate, primary peritoneal and ovarian cancers by the US Food and Drug Administration (FDA) and the European Medicines Agency (EMA) [134, 135]. Veliparib has not been approved for clinical use and is currently being studied in combination with chemotherapy or targeted therapies for solid tumors, such as NCT02032277, NCT02152982, NCT02163694, and NCT02264990. Several novel inhibitors such as Pamiparib and Fluzoparib have been developed in the past few years. However, their advantages still need to be further validated and designed to evaluate Pamiparib in recurrent ovarian cancer (Phase III, NCT03519230) and advanced gastric cancer (Phase II, NCT03427814) are in progress. The initial efficacy of PARP inhibitors is positive, but after long-term application, patients will develop resistance to them, leading to tumor progression. The main mechanisms may include: The expression of drug efflux transporter ABCB1 increased [136, 137], the change of action target PARP [138, 139], and the recovery of Breast cancer susceptibility gene 1/2 (BRCA1/2) function [140, 141]. There are many promising approaches to overcome resistance to PARPi, such as enhancing the antitumor effects of PARPi, acquiring vulnerability to PARP inhibitor-resistant cancers, and delaying the emergence of resistance by inhibiting mutant phenotypes [142]. Here we mainly introduce the combined application of immunotherapy and PARPi.

As a DNA damage sensor, PARP can help cells maintain the ability to stop replicating and repair DNA, while the homologous recombination repair function is defective in BRCA mutant cells of breast cancer. The loss of PARP function will lead to the accumulation of broken DNA and the ultimate cell death [143, 144]. This is the basic mechanism of PARPi in treating BRCA1/2 defect cancers. In addition, DNA repair defects activate the immune system by affecting the expression of relevant immune molecules, such as BRCA-deficient and homologous recombination deficiency (HRD) ovarian cancer presenting with new antigen formation and increased infiltration of CD3^+^ and CD8^+^ immune cells. Tumors with BRCA1/2 mutations also showed increased expression of PD-L1 and PD-1 in intraepithelial and peritumoral immune cell compartments [145]. On the other hand, damaged DNA can activate the innate immune system. The cyclic GMP-AMP synthase (cGAS)-STING pathway is an important mechanism to protect cells from cytoplasmic DNA damage, and the anti-tumor efficacy of PARP inhibition depends on the activation of the STING pathway. In BRCA deficiency [146] and BRCA wild-type models [147], this antitumor activity was attenuated due to the loss of cytotoxic T cells or the loss of signaling in STING pathways. One study showed that PARPi induced upregulation of inflammatory factors in BRCA mutants through the cGAS/STING pathway, and in the wild type, it may induce mutations in related genes to express MHC class 1 neoantigen with strong affinity, which is consistent with the above two mechanisms. It was also suggested that PARPi treatment could induce the above two types of breast cancer response to immunotherapy [148]. Recent studies have shown that the immunomodulatory effect of PARPi can help to enhance the therapeutic effect. PARPi treatment leads to the accumulation of dsDNA in the cytoplasm, which activates the cGAS-STING-TBK1-IRF3 innate immune pathway, enhances type I IFN response, increases the number of tumor-infiltrating lymphocytes and induces the immunogenic microenvironment (Fig. 3). In addition, PARPi increased the number of PD-L1 cells, and further studies confirmed that the combination of immune checkpoint blockade and PARPi (e.g., olaparib) increased the percentage of tumor-infiltrating CD8^+^ cells, suggesting the possibility that this combination therapy could synergically limit tumor growth and prolong survival[146, 147, 149]. At the same time, relevant studies have shown that this combination therapy is also effective in solid tumors, and this phenomenon is more evident in HR-deficient TNBC [150, 151].

These data suggest that by interfering with HR repair, PARP inhibitors may increase neoantigen production and stimulate tumor immune microenvironment, thus synergistic with immune checkpoint inhibitors. A Phase I/II clinical trial is currently underway to evaluate the safety and efficacy of Niraparib and pembrolizumab in combination therapy for patients with advanced or metastatic triple-negative breast cancer or recurrent ovarian cancer (NCT02657889). The results of the ovarian cancer cohort showed an ORR value of 18% and a disease control rate of 65% in patients with Phase 1 and 2 ovarian cancer, indicating that this combination has good antitumor activity in patients with ovarian cancer [152]. The combination of olaparib and ciladinib, a VEGFR inhibitor, is superior to olaparib monotherapy in relapsed platinum-sensitive ovarian cancer [153]. A phase III clinical study is also underway in combination with a PARP inhibitor (Niraparib), VEGFR inhibitor (Bevacizumab), and an immune checkpoint inhibitor (TSR-042) to treat platinum-resistant ovarian cancer (NCT03574779).

## CD38 monoclonal antibody

As mentioned earlier, overexpression of CD38 is often viewed as a malignant phenotype, and the mechanisms of CD38 mab against CD38 + cancer cells are diverse. Including complement-dependent cytotoxicity, antibody-dependent cellular cytotoxicity, antibody-dependent cellular phagocytosis, programmed cell death, modulation of enzymatic activity, and immunomodulatory activity [154]. Part of the action may be mediated by NK cells and monocytes [155]. Daratumumab (Dara) is a monoclonal antibody against CD38 mediated by human immunoglobulin G1 kappa (IgG1κ). It was approved by the FDA in 2015 as a monotherapy for multiple refractory myelomas and by the European Medicines Agency (EMA) in 2016 [156, 157].

The use of alemtuzumab significantly prolonged the overall survival of MM patients, and the effect was better when combined with lenalidomide and dexamethasone. The adverse reactions mainly included neutropenia, thrombocytopenia, and anemia, and the reason for disease recurrence in some patients may be that the minimal residual lesions were not eliminated [158]. In addition, daratumumab has some efficacy against CLL and enhances the anti-CLL activity of ibrutinib by participating in the regulation of the BCR signal [159]. Isatuximab (Isa)/SAR650984 is another CD38 monoclonal antibody in clinical development that induces apoptosis without the addition of external cross-linking agents and may inhibit the ADP-ribosyl cyclase activity of CD38 via allosteric antagonists [160]. It also significantly killed MM cells with high expression of CD38 by inducing isotype aggregation, leakage of lysosomal associated cathepsin B and lysosomal associated membrane protein-1 (LAMP-1), and production of reactive oxygen species [161]. Targeting CD38 with Isa induces immunomodulation, alleviating immunosuppression by inhibiting proliferation and migration of regulatory T cells and enhancing anti-MM immunity mediated by CD8^+^ T cells and NK cells. Moreover, this effect is enhanced in combination with Pomalidomide (Pom)/Len [93]. In addition to a single antibody targeting CD38, a novel anti-CD38 /CD3 bi-specific T cell recruitment antibody AMG-424 has been discovered in recent years, which can kill cancer cells expressing high and low levels of CD38 in vitro and trigger T cell proliferation, and induce tumor growth inhibition and consumption of peripheral B cells in vivo. Without inducing excessive cytokine release, this optimized T-cell recruitment antibody may have significant clinical activity in MM patients [162]. CD38-specific CAR-T cell therapy has also been shown to be effective in vitro and in vivo against hematological malignancies such as myeloma and B-cell non-Hodgkin’s lymphoma [163, 164].

## The combination therapy

In the tumor microenvironment, various enzymes involved in the NAD metabolic pathway are closely related to each other. Inhibition of NAMPT to limit the production of NAD and activation of NAD consumption enzyme to enhance the consumption of NAD. Combining these two methods can significantly reduce the production of NAD in tumor cells and enhance anti-tumor efficacy. For example, studies have shown that SIRT-1 is the main consumer of NAD^+^ in IDH mutant glioma. Activation of SIRT-1 by genetic or pharmacological means can promote NAD^+^ depletion and enhance the effect of NAMPTi, and this combination therapy can reduce chemotherapy-induced cytotoxicity [59]. The combination of PARPi and NAMPTi is an effective method to inhibit NAD^+^ production in Ewen’s sarcoma. The mechanism includes depletion of NMN and NAD, reduction of PAR activity, DNA damage, and increased apoptosis. This combination does not add cytotoxicity. Moreover, it can lead to tumor regression, delay disease progression and improve survival rate, which is a potential therapeutic strategy [165]. Currently, the clinical efficacy of NAMPT inhibitors is limited, partly due to the development of drug resistance caused by alternative NAD^+^ production pathways in tumors. Studies have shown that the NAPRT gene, the second NAD^+^ production enzyme, is amplified and overexpressed in cancers, and the combined inhibition of NAMPT and NAPRT may help improve drug resistance in tumors [112].

Tumor immunotherapy, including immune checkpoint inhibitors, adoptive cell therapy, and tumor vaccines, has been widely used in clinical applications. The compounds related to NAD pathways play a role in the tumor immune microenvironment; they can change the tumor cells of the immune microenvironment and regulate the expression of cytokines through the related signal pathways, consequently affecting the function of immune cells. This suggests that NAD pathways related to tumor immunotherapy are also possible. Lv Hongwei et al. have shown that the reduction of NAD^+^ in tumor-infiltrating T cells will affect their anti-tumor effect [56]. Further studies by Wang Yuetong et al. showed that NAD^+^ supplementation could further enhance the efficacy of tumor clearance in CAR-T and anti-PD-1 therapy models. NAD^+^ supplementation can enhance the anti-tumor effect of T-cell-based immunotherapy, but whether it can be used as a treatment strategy still needs to be further verified in clinical trials[57].

All-trans retinoic acid (ATRA) and IFN-β can induce up-regulation of CD38 in tumor cells, and CD38 inhibits CD8^+^ T cell function through adenosine receptor signaling, thereby making tumor resistant to PD-L1/PD-1 blockade. CD38 or adenosine receptor blockade is an effective strategy to overcome drug resistance. This has also been verified in animal models of lung cancer[166]. On the one hand, inhibition of CD38 expression on the surface of Th1/17 cells can improve the metabolic adaptability of T cells by enhancing oxidative phosphorylation and glutamine catabasis. On the other hand, it can affect the NAD-SIRT1-FOXO1 axis by increasing NAD, thus boosting the anti-tumor potential of hybrid Th1/17 cells. Strategies targeting the CD38-NAD axis can improve the efficacy of anti-tumor adoptive T cell therapy [167]. Studies have shown that CD38 can enhance adenosine (ADO) content in the tumor microenvironment to promote tumor metastasis and inhibit CD8^+^ T cell function while inhibiting CD38 or blocking ADO receptors can effectively improve the efficacy of targeted immune checkpoint therapy [166, 168]. Tumor-infiltrating CD38^+^ CD8^+^ T cells were significantly less capable of secreting IFN-γ and granzyme B than their adjacent normal tissues [169], and negative costimulatory markers PD-1 and CD101 were also increased in this subpopulation. However, CD8^+^ T cells expressing CD38 in the tumor microenvironment still have strong cytokine secretion and tumor-killing ability, partly due to the high expression of CD103 in CD38^+^ CD8^+^ T cells, which helps to increase the resident capacity of immune cells in tumor tissues. Moreover, CD103^+^ CD38^+^ CD8^+^ T cells were blocked by PD-1 in vitro and secreted higher levels of TNF-α to mediate anti-tumor immune response [170].

Radiation therapy, a traditional cancer therapy, has also been shown to improve anti-tumor efficacy in combination with NAMPTi or PARPi. Studies have shown that NAMPT inhibitor GMX1778 can enhance the efficacy of 177Lu-dotatate in neuroendocrine tumors, indicating that it can be used as a radiosensitizer. For its mechanism, the researchers speculate that the ionizing radiation released by 177Lu can cause DNA damage, thus activating PARP-1. Moreover, the inhibition of Gmx1778 on the NAD + remedy pathway reduces the level of NAD^+^ to the lethal level, consistent with the results of in vitro experiments [171]. In colorectal cancer models, radiotherapy combined with the PARP inhibitor Veripanil enhances the expression of tumor surface antigen MHC-1, cytokines INF-β and CXCL10, and PD-L1, thereby enhancing the anticancer efficacy of immune checkpoint inhibitors[172].

## Other novel treatments

**Fig. 5:**
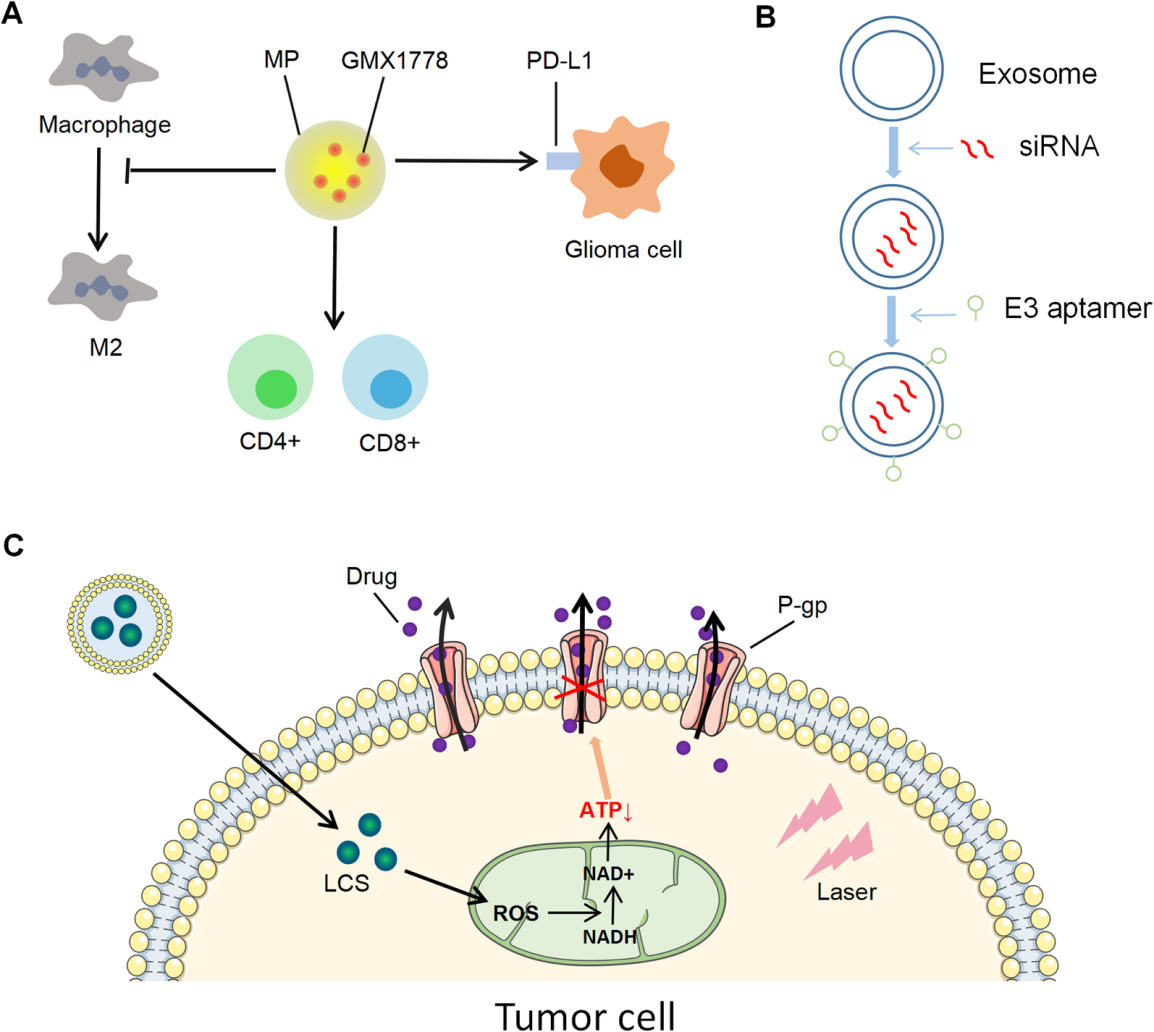
New types of therapy.** A** Microparticle MP can deliver NAMPT inhibitor GMX1778 to glioma cells in A directional manner and control its release amount to resist tumor by up-regulating PD-L1 expression, inhibiting m2-type macrophage generation, and collecting CD4 + and CD8 + T cells. **B** Encapsulation of exosomes protects siRNA from degradation, and modification of E3 aptamer can be directed to prostate cancer cells, and siRNA plays an anti-tumor role by silencing SIRT-6. **C** After the hybrid nanoparticles of silicon carbide (LSC) coated by lipid membrane enter the cell, LCS directionally binds to mitochondria and generates ROS under infrared light. ROS oxidizes NADH to NAD + and reduces ATP production, inhibiting p-glycoprotein (P-GP) from excreting chemotherapeutic drugs out of the cell, thus improving tumor drug resistance

In recent years, the integration of medicine and industry, multi-disciplinary interaction and the progress of new materials have also put forward some new ideas and methods for tumor therapy. Researchers increasingly favor some small molecules with unique physical and chemical properties and targeting functions. For example, microparticles (MP) loaded with NAMPT inhibitors can directly act on brain tumors and control their continuous release in situ, thus reducing the toxicity of NAMPTi to normal cells [173]. Further studies found that GMX1778, a NAMPT inhibitor mediated by particles, can change the tumor immune microenvironment of GBM in mice. It is characterized by up-regulation of immune checkpoint PD-L1, recruitment of CD3^+^, CD4^+^, and CD8^+^ T cells, and reduction of M2-polarized immunosuppressive macrophages (Fig. 5A). These changes, combined with PD-1 checkpoint blockade in vivo, significantly improve the survival rate of GBM-carrying animals, which is a safe and effective method to enhance GBM immunotherapy [174]. E3 aptamer conjugates exosomes on the surface to protect siRNA from degradation and target siRNA to prostate cancer cells, thereby silencing SIRT-6, promoting cancer cell invasion and metastasis in vivo (Fig. 5B). This approach is expected to replace traditional drugs for the treatment of advanced prostate cancer [175]. As a drug delivery system, nanoparticles have the advantage of improving bioavailability and reducing drug metabolism and side effects [176]. Liposomes are considered good nanocarriers because of their similar morphology to cell membranes and ability to bind hydrophilic compounds [177]. Talazoparib delivered by nanoliposomes in BRCA-deficient mice improves efficacy, delays tumor progression, and prolongs survival with fewer side effects [178]. Other research groups have identified lipid membrane-coated hybrid silicon carbide (LSC) nanoparticles that target mitochondria via pyruvate and produce ROS in mitochondria under near-infrared (NIR) laser irradiation to oxidize NADH to NAD^+^. To reduce the amount of ATP available at efflux pumps. This method also reduced efflux pump expression and increased intracellular distribution (Fig. 5 C). In vivo data showed that the combination of drug-loaded LSC nanoparticles and NIR laser can effectively inhibit the growth of multidrug resistant tumors without significant systemic toxicity [179].

FK866 loaded Bismuth-Humic Acids heterojunction (BI-HA /FK866) is a novel strategy to enhance photocatalytically induced NAMPT regulatory therapy, improving electron-hole pair separation by BI-HA heterojunction structure. Under mild near-infrared irradiation, BI-HA /FK866 can effectively induce ROS production and GSH consumption, which can induce DNA damage of tumor cells, thereby increasing sensitivity to NAMPT inhibitor (FK866), down-regulating NAD/ERK/NF-κB signaling pathway, and ultimately preventing cancer progression. This provides a new approach to solving the problem of cancer recurrence and migration caused by penetration depth limitation [180]. Antibody-drug conjugates (ADCs) are a treatment approach in which NAMPT inhibitors are targeted as drugs to cancer cells to achieve their killing effect. This reduces the hematological, retinal, and cardiac toxicity of NAMPTi’s systemic effects, improving the treatment window for NAMPTi. It has considerable clinical application prospects [181]. Previous examples of ADCs applied to NAMPTi, such as nampti-adc designed by Karpov et al. and Neumann et al. [182], although these drugs have effective in vivo and in vitro activity, the problems of selectivity and toxicity can be further optimized. Recently, Niels Bohnke et al. used the conjugation strategy to optimize the scaffold and joint structure of ADCs to produce ADCs with low aggregation, high efficiency and selectivity and proved that NAMPTi could serve as a practical payload class in vitro and in vivo experiments. ADCs are designed to treat solid and hematologic tumors [183]. All the new therapeutic methods introduced above can assist related drugs in acting on tumor cells, and they have unique physical and chemical properties, strong targeting ability, compatibility, and small side effects. In conclusion, combining new technologies is a new opportunity and challenge with broad application prospects to improve the efficacy of new therapies for cancer further.

## Discussion and prospect

With the deepening of relevant studies in recent years, the role of the NAD metabolic pathway in the tumor immune microenvironment is becoming clearer; some key enzymes play an essential role in tumorigenesis and cancer development. In addition, some drugs have entered clinical trials with good results (Table 1). First of all, due to the characteristics of high metabolism in the tumor microenvironment, NAMPT, a rate-limiting enzyme of NAD synthesis, is generally considered to play a major role in promoting cancer. However, some studies have found that NAMPT can also inhibit tumor migration and metastasis by regulating SIRT-1, indicating that the mechanism remains to be further explored. In recent years, many NAMPT inhibitors have entered the stage of clinical trials, and their efficacy in related tumors has been gratified, but their non-selective cytotoxicity has become a major difficulty in clinical application. We believe optimizing the drug is still the main solution to the problem structure to make it more targeted or add exogenous NAD supplements to reduce its side effects. Sirtuins family is a large family with complex functions. As mentioned above, their role in the tumor microenvironment is not absolute, and they play different roles in different tumors, which is also one of the difficulties in developing sirtuins targeted anticancer drugs. In recent years, although it has been proved that some bioactive substances can exert anticancer effects by regulating sirtuins and there have been many structural innovations in developing sirtuins drugs, there is still a long way to use sirtuins in the clinic. However, the role of PARP and CD38 in the tumor immune microenvironment is more precise and related PARP inhibitors and CD38 mab have been applied in the clinic and achieved good efficacy. The current research direction is mainly to improve the generation of drug resistance, enhance efficacy, and expand the scope of tumor application.

**Table 1:** Some drugs are already in clinical trials.

Treatment	Phase	Tumor types	Efficacy	ClinicalTrials.gov Identifier
Talazoparib	III	Advanced and/or metastatic breast cancer with BRCA mutation	PFS: 8.6 months	NCT01945775
Niraparib + Pembrolizumab	I/II	Ovarian Cancer/TNBC	ORR: 15.1%/18.2%	NCT02657889
Niraparib + Pembrolizumab	II	NSCLC	ORR: 56.3%	NCT03308942
Pamiparib + Tislelizumab	I/Ib	TNBC with either known germline or somatic BRCA1/2 mutations or with documented HRD	ORR: 47.4% PFS: 8.4 months	NCT02660034
Daratumumab	II	Follicular Lymphoma	ORR: 12.5% PFS: 3.3 months	NCT02413489
Isatuximab + Cemiplimab	I/II	Metastatic castration-resistant prostate cancer (mCRPC) or advanced non-small cell lung cancer (NSCLC)	lack of efficacy	NCT03367819
OT-82	I	Relapsed or Refractory Lymphoma	No results	NCT03921879
ATG−019	I	Solid Tumor, Non-Hodgkin’s Lymphoma	No results	NCT04281420
KPT−9274	I	Acute Myeloid Leukemia	No results	NCT04914845

PARP inhibitors: Talazoparib, Niraparib, Pamiparib; PD-L1 antibody: Pembrolizumab; PD-1 antibody: Tislelizumab, Cemiplimab; CD38 monoclonal antibody: Isatuximab; NAMPT inhibitor: OT-82, ATG-019,KPT-9274

*TNBC* Triple-negative breast cancer,* NSCLC* non-small cell lung cancer,* HRD* Homologous recombination defect,* PFS* progression-free survival,* ORR* objective response rate

Finally, associated combination therapies and therapies based on emerging technologies are more effective approaches than monotherapy. The functions of NAMPT, Sirtuins, PARPs, and CD38 enzymes in the NAD pathway are mutually synergistic and interactive. For example, NAMPT can inhibit SIRT-1 from preventing tumor migration and metastasis, while SIRT-1 can promote the consumption of NAD and enhance the role of NAMPTi. This provides a theoretical basis for the combination therapy of these drugs. Currently, the combination scheme, mechanism research and efficacy evaluation of the drug combination are still relatively scarce, partly because of the lack of corresponding drugs, but the drug combination still has considerable research value and application prospects. Based on the unique role of enzymes related to the NAD pathway in the tumor immune microenvironment, the combination of tumor immunotherapy and related drugs will play a “1 + 1 > 2” role. For example, the efficacy of olaparib or CD38 mab combined with immune checkpoint blocking therapy has been confirmed. The selection of this combination is a good method to improve tumor efficacy and drug resistance. In recent years, some tumor therapies based on nanoparticles, exosomes, lasers, and other new technologies are also gaining traction. These adjuvant methods reduce the side effects of drugs, enhance treatment targeting, and improve efficacy. The combination of these new technologies, the development of basic research and the proposal of new ideas are the great impetus for cancer treatment progress. It is necessary to actively promote the progress of these therapies so that they can bring benefits to cancer patients as soon as possible.

## Data Availability

The materials that support the conclusion of this review have been included in the article.

## Abbreviations

APCs: Antigen-presenting cells
BRCA: Breast cancer susceptibility gene
cGAS: Cyclic guanosine monophosphate-adenosine monophosphate synthase
CLL: Chronic lymphocytic leukemia
CTLs: Cytotoxic T cells
CXCR4: Chemokine (C-X-C motif) receptor 4
DAMP: damp-related molecular pattern protein
EGFR: Epidermal growth factor receptor
EMT: Epithelial-mesenchymal transformation
EGFR: Epidermal growth factor receptor
eNAMPT: Extracellular NAMPT
GBM: Glioblastoma multiforme
GBM: Glioblastoma multiforme
GSC: Glioma stem cell
HCC: Hepatocellular carcinoma
HDAC: Histone deacetylase
HIF-1: Hypoxia-inducible factors-1
IL: Interleukin
iNAMPT: Intracellular NAMPT
iNOS: Inducible nitric oxide synthase
IFN-γ: IRF1:Interferon regulatory factor-1
MAPK: Mitogen-activated protein kinase
MM: Multiple myeloma
MMP9: Matrix metalloproteinase 9
MDSC: Myeloid-derived suppressor cell
NAD: Nicotinamide adenine dinucleotideNAMPT:NAMPTi:NAMPT inhibitor
NAMPT:NAMPTi: NAMPT inhibitor
NAMPT/NAPRT: Nicotinate phosphoribosyltransferase
NF-κB: Nuclear factor kappa-B
NO: Nitric oxide
NSCLC: Non-small cell lung cancer
PD-L1: Programmed death ligand-1
PH pathway: Preiss-Handler pathway
PROTAC: Proteolysis-targeting chimera
ROS: Reactive oxygen species
STAT1: Signal transducer and activator of transcription 1
STING: Stimulator of interferon genes
TCR: T-cell receptor
TIL: Tumor-infiltrating lymphocyte
TKI: Tyrosine kinase inhibitor
TLR4: Toll-like receptor 4
TNBC: Triple-negative breast cancer
TNF: Tumour necrosis factor
Tregs: Regulatory T cells
TRPM2: Transient receptor potential M2
KEAP-1: Kelch-like ECH-associated protein 1
